# Seroprevalence of immunity to hepatitis A and hepatitis B among gay, bisexual and other men who have sex with men (GBMSM) attending sexual health clinics in London and Leeds, England, 2017–2018

**DOI:** 10.1136/sextrans-2024-056134

**Published:** 2024-06-26

**Authors:** Rachel Roche, Ruth Simmons, Hester Allen, Megan Glancy, Anca-Maria Balan, Maria Bolea, Ross Harris, Monica Desai, Hamish Mohammed, Caroline Sabin, Samreen Ijaz, Sema Mandal

**Affiliations:** 1 Blood Safety, Hepatitis, Sexually Transmitted Infections (STI) and HIV Division, UK Health Security Agency-Colindale, London, UK; 2 The National Institute for Health Research Health Protection Research Unit in Blood Borne and Sexually Transmitted Infections at University College London in partnership with the UK Health Security Agency, London, UK; 3 Virus Reference Department, UK Health Security Agency-Colindale, London, UK; 4 Centre for Clinical Research, Epidemiology, Modelling and Evaluation, Institute for Global Health, University College London, London, UK

**Keywords:** HEPATITIS A, HEPATITIS B, Seroepidemiologic Studies, VACCINATION, Prevalence

## Abstract

**Objectives:**

Although hepatitis A virus (HAV) and hepatitis B virus (HBV) immunisation is recommended in the UK for gay, bisexual and other men who have sex with men (GBMSM), data on immunisation coverage are limited. We aimed to determine the seroprevalence of HAV and HBV immunity among a sample of GBMSM attending sexual health services (SHS) in England.

**Methods:**

Residual serum samples from HIV/syphilis testing for adult GBMSM attending eight SHS in London and one in Leeds were tested for markers of HAV immunity (HAV IgG) and HBV immunity (anti-HBs) using an unlinked anonymous approach. We estimated seroprevalence of HAV and HBV immunity overall and stratified by individuals’ characteristics, which we obtained from the Genitourinary Medicine Clinic Activity Dataset Sexually Transmitted Infection (STI) Surveillance System. We used logistic regression to calculate crude and adjusted ORs between seropositivity and demographic and clinical characteristics.

**Results:**

Seroprevalence of immunity to HAV (74.5% of 2577) and HBV (77.1% of 2551) was high. In adjusted analysis, HAV IgG seroprevalence varied by clinic and WHO region of birth (global p<0.001 for each), increased with older age (ORs of 1.50 (95% CI 1.18 to 1.86), 2.91 (2.17 to 3.90) and 3.40 (2.44 to 4.75) for ages 26–35, 36–45 and >46 vs 18–25 years (global p<0.001), was higher in those with an STI in the past year (1.58 (1.25 to 2.00); p<0.001) and those who were living with HIV (1.82 (1.25 to 2.64); p<0.001). Anti-HBs seroprevalence varied by clinic (global p<0.001), increased with older age (global p<0.001) and was higher in those with an STI in the past year (1.61 (1.27 to 2.05); p<0.001).

**Conclusion:**

Our findings provide a baseline seroprevalence from which to monitor serial levels of immunity to HBV and HAV in GBMSM accessing SHS. Levels of immunity for both viruses are high, noting samples were taken after recent widespread outbreaks and vaccination campaigns. High vaccine coverage in all GBMSM should be maintained to prevent further outbreaks.

**WHAT IS ALREADY KNOWN ON THIS TOPIC**

- Immunisations for hepatitis A virus (HAV) and hepatitis B virus (HBV) are effective and are recommended in the UK for gay, bisexual and other men who have sex with men (GBMSM); however, data on HAV and HBV immunisation coverage among GBMSM in the UK are limited. Serial seroprevalence surveys allow monitoring immunity in this population. Outbreaks of both infections among GBMSM in recent years highlighted variation in vaccine provision by clinics.

**WHAT THIS STUDY ADDS**

- Our study found high prevalence of immunity through infection or immunisation to HAV (74.5%) and HBV (77.1%) in this sample of GBMSM attending sexual health services (SHS) located mainly in London, England.

**HOW THIS STUDY MIGHT AFFECT RESEARCH, PRACTICE OR POLICY**

- This study provides a baseline seroprevalence from which to monitor serial immunity to HBV and HAV infection in GBMSM accessing SHS. High vaccine coverage in all GBMSM should be maintained to prevent further outbreaks.

## Introduction

Hepatitis A virus (HAV) and hepatitis B virus (HBV) are viruses which infect the liver and can cause acute liver disease; HBV can also lead to chronic infection. Both infections have low incidence in the UK; around 300–400 cases of HAV were reported annually in England and Wales between 2010 and 2015 and reported acute HBV cases have continued to decrease from 513 in 2011 to an average of 350 in 2015–2021.^1 2^ However, certain populations including gay, bisexual and other men who have sex with men (GBMSM) are at increased risk of infection.^1 3^ Safe and effective vaccines for both viruses have been available since 1995, and national clinical and public health guidelines recommend testing and vaccinating individuals at risk of infection, including GBMSM, when they attend sexual health services (SHS) and other healthcare settings.^4 5^

In 2016–2018, there was a sustained HAV outbreak among GBMSM in the UK, which was linked to other outbreaks across Europe.^6–8^ Acute HBV outbreaks also occur and since the 1990s, the A2 strain (HBV prisoner variant) has frequently been identified in outbreaks associated with injecting drug use and sexual transmission including among GBMSM in England.^9 10^ In the past decade, this variant was associated with regional outbreaks of HBV among GBMSM who identify as heterosexual (*HI*-MSM)—a group at high risk of HAV, HBV and other sexually transmitted infections (STIs) who may not access services targeted to gay or bisexual identifying GBMSM.^11^ Prior to the 2016–2018 HAV outbreak, commissioning and implementation of HAV vaccination varied between SHS and over time within individual SHS.^12^ In 2017, in response to the outbreak, clearer guidance on opportunistic HAV vaccination of all GBMSM attending SHS was issued by the British Association of Sexual Health and HIV (BASHH).^5^ In contrast, HBV vaccination of GBMSM has been more consistently commissioned locally as a routine, core service in SHS. In 2017–2018, all HAV and HBV immunisation programmes were threatened by severe constraints in HBV and HAV vaccine supply due to global manufacturing issues, which led to prioritisation of scarce vaccine stock that impacted vaccination of GBMSM in SHS.^13 14^

Monitoring of HAV and HBV vaccine uptake, susceptibility and immunity in GBMSM attending SHS is needed to evaluate the implementation and impact of immunisation programmes, inform modelling to assess risk of future outbreaks and support vaccine prioritisation decisions in the event of vaccine shortages. Data on receipt of HAV and HBV vaccines are recorded in GUMCAD (Genitourinary Medicine Clinic Activity Dataset), the mandatory surveillance system for STIs in England,^15^ but an audit showed that there was a high degree of under-reporting of previous immunity by SHS, leading to an overestimation of the number of people assumed to be eligible for vaccination and resulting in artefactually low vaccine coverage among GBMSM.^16^ Continued efforts are being made to improve the reporting of immunity to this dataset.

Serosurveys provide information on population levels of immunity; there have been small serosurveys of HAV and HBV in individual SHS, but none have been sufficiently representative to make inferences about population-level immunity and susceptibility in GBMSM from which to monitor trends and inform national policy recommendations. This study, therefore, aimed to undertake a representative serosurvey across multiple sites to (1) estimate the seroprevalence of HAV and HBV immunity in a sample of GBMSM attending geographically distinct SHS, and (2) investigate the association of HAV and HBV immunity with demographics and indicators of risk.

## Methods

### Data collection and management

#### Serum samples

Five of seven invited diagnostic testing laboratories agreed to participate, providing samples from eight SHS in London and one in Leeds. Serum samples from attendees who self-identified as GBMSM on their attendance (aged ≥18 years) having blood taken for HIV and/or syphilis testing at participating SHS between April 2017 and December 2018 were included. Residual blood samples were identified using three different methods specified by local diagnostic laboratory and SHS staff: (1) Public Health England (PHE) generated a sample list of attendees recorded as GBMSM in GUMCAD using patient identification codes from the GUMCAD STI Surveillance System, a pseudonymised and depersonalised patient-level dataset of all services provided and STI diagnoses made at publicly commissioned SHS in England,^15^ and shared this with the participating laboratory for sample retrieval; (2) the laboratory identified samples by linking between their laboratory and electronic patient record management databases; and (3) SHS clinical staff identified and flagged samples for retrieval in the laboratory.

Residual serum samples were sent to the Blood-Borne Virus Unit of the Virus Reference Department at PHE Colindale for testing. One sample per individual was included in the analysis; if there were multiple samples for an individual, the sample and data from their most recent attendance were retained.

### Unlinking, anonymising and data linkage

All samples were unlinked and anonymised before testing. Variables of interest were obtained from GUMCAD. A new study ID was created, and original identifiers were stripped from samples and GUMCAD demographic and risk data before testing.

### Data fields and definitions

Included GUMCAD variables were demographics (age, country of birth); infection history (history of STI within the past year, HAV diagnosis, HBV diagnosis, HIV status); vaccine history (HAV dose 1, 2; HBV dose 1, 2, 3, 4, booster); and immunity (HAV or HBV immune via self-reported or documented vaccination or natural infection). WHO region of birth was derived from self-reported country of birth, with the UK considered separately from the rest of Europe.^17^ Samples were excluded if there were insufficient data to link to a GUMCAD attendance or no corresponding record in GUMCAD. A minimum dataset of the same GUMCAD variables was created for all other GBMSM attending the clinics during the sampling period for comparison (definitions in online supplemental table 1).

10.1136/sextrans-2024-056134.supp1 Supplementary data

### Sample size

To determine target sample sizes, we calculated the 95% CIs of seroprevalence estimates by age group for each clinic group. Age was grouped as <40 years, 40–59 years and ≥60 years, based on disease risk and consideration of possible age-based vaccination strategies. General population HAV seroprevalence estimates were used in the calculations because no suitable GBMSM-specific seroprevalence estimates were available.^18 19^ Sample sizes were limited in practice by (1) resource and clinic/laboratory capacity to collect samples and (2) the number of GBMSM attending SHS for an HIV and/or syphilis test, so attendance data were also considered. Sample sizes of 260 in the <40 years age group, 250 in the 40–59 years age group and 150 in the 60+ years age group were used as the target, giving 95% CI of ±5% in <40 years age group, ±6% in 40–59 years age group and ±8% in 60+ years age group (online supplemental table 2). For the 60+ years age group, however, numbers were likely to be lower in all but the largest London clinics. This was deemed acceptable as transmission and force of infection in older age groups are small because these individuals are mostly immune and so not a priority in vaccination considerations.

Hepatitis B surface antibody (anti-HBs) testing was added to the study after completion of sample collection, with subsequent analysis determined by the numbers of samples available and the anti-HBs prevalence found.

### Serological analysis

Serological testing for antibodies to HAV (anti-HAV IgG) and HBsAg (anti-HBs) was undertaken on the Architect platform (Abbott, Maidenhead, UK) in accordance with the manufacturers’ instructions.

Immunity to HAV, indicative of past or recent infection or vaccination, was defined using a sample/cut-off ratio of ≥1.0 to classify a sample reactive for HAV IgG.

Immunity to HBV, indicative of vaccination or past exposure, was defined by measuring anti-HBs titre against a generated calibration curve with samples with concentration values of ≥10.0 mIU/mL considered reactive.

### Data analysis

Clinics were grouped by laboratory of testing as some were small satellite clinics, resulting in five clinic groups for analysis. We described the demographic and risk characteristics of our sample and compared these with the characteristics of other GBMSM attending the participating clinics during the sampling period using χ^2^ tests.

Evidence of HAV immunity (HAV IgG), HBV immunity (anti-HBs) and combined HAV/HBV immunity was calculated for the overall sample and by age group, region of birth, clinic group, HIV status, past STI and GUMCAD-recorded HAV/HBV vaccination (‘any vaccine dose’ of either vaccine derived from individual dose variables), diagnosis and immunity. The χ^2^ test was used to test for differences between groups. Weighted seroprevalence of HAV and HBV immunity was calculated using the *svyset* command in STATA, using clinic group as the primary sampling unit and weighting for factors which were strongly associated with seroprevalence and for which the distribution significantly differed between our sample and other GBMSM attendees.

Logistic regression was used to identify factors associated with HAV or HBV immunity as crude and adjusted ORs with 95% CI, using the following variables: age group, clinic group, past STI (history of STI in the past year), HIV status and WHO region of birth. Variables were tested for inclusion in the final model using the Wald test and included if significant (p<0.05); for categorical variables, this was specified as a joint test of all indicator variables being equal to zero. Two-way interactions with age group and clinic group were also examined via joint Wald tests. The resulting p values represent the overall impact of the variable on the model.

## Results

### Sample characteristics compared with other GBMSM clinic attendees

Sampling data and processing laboratory for each clinic group are presented in table 1.

**Table 1:** Sampling details for sentinel sites

Laboratory	Clinics	Location	Attendance dates of sample collection	No of samples	Total GBMSM attendees at clinic over time period of sample collection	% of clinic population sampled
The Doctor’s Laboratory North London	Central Middlesex Hospital, Northwick Park Hospital, Tudor Centre	London	01 Nov 2017–28 Dec 2017 and 02 Jul 2018–28 Sep 2018	450	995	45.2
Homerton Hospital	Homerton Hospital	London	09 Jan 2018–11 Jul 2018	566	2007	28.2
Imperial/St Mary’s Hospital	Jefferiss Wing Centre for Sexual Health	London	06 Aug 2018–17 Oct 2018	467	1200	38.9
St Thomas’ Hospital	Burrell Street Sexual Health Clinic, Streatham Hill Primary Health Care Centre, Walworth Sexual Health Clinic	London	01 Oct 2018–31 Dec 2018	643	3357	19.2
Leeds Teaching Hospitals Trust	Leeds Sexual Health	Leeds	01 Apr 2017–13 Jul 2017	451	969	46.5

GBMSM, gay, bisexual and other men who have sex with men.

A total of 2577 serum samples were tested for HAV IgG; 2551 had sufficient blood remaining and were also tested for anti-HBs. Over half (1504 of 2577, 58%) of the study sample were aged less than 36 years, with 18% (473 of 2577) aged 46 years and over. Just over half (52.9%) were UK born. Age and country of birth distribution were both similar between our sample and other GBMSM attendees. In our sample, those who had a past STI in the last year and those were living with HIV were lower compared with other GBMSM attendees (p<0.01). The proportion with recorded HAV vaccination was similar between our sample and other GBMSM attendees, but the proportions with recorded HBV vaccination, and recorded HAV and HBV immunity were higher in our sample (online supplemental table 3).

### Weighted HAV IgG seroprevalence and associated risk factors

The weighted seroprevalence of HAV IgG was 74.3%. Overall, 75% (1919 of 2577) of samples were HAV IgG positive, indicating immunity due to past exposure to HAV infection or vaccination (table 2). HAV immunity varied by clinic group, ranging from 59.7% in Leeds to 84.6% in London St Mary’s Hospital (global p<0.001). In multivariable analysis, seroprevalence of HAV immunity increased with older age (ORs of 1.50, (1.18 to 1.86), 2.91 (2.17 to 3.90) and 3.40 (2.44 to 4.75) for ages 26-35, 36-45 and >46 vs 18-25 years (global-p<0.001), and was higher in those with a past STI compared with none (OR 1.58, 1.25 to 2.00) and higher among those living with HIV compared with those without (OR 1.82, 1.25 to 2.64) (table 3). By region of birth, HAV immunity was most prevalent among those born in the African region (OR 4.73, 1.68 to 13.31), and less prevalent among those born in the rest of Europe (OR 0.56, 0.44 to 0.71) and the Americas (OR 0.64, 0.45 to 0.91) compared with those born in the UK (p<0.01).

**Table 2:** Seroprevalence of immunity to hepatitis A infection, hepatitis B infection and combined immunity to hepatitis A and B infection, by clinic group and patient characteristics

Characteristic	HAV IgG				Anti-HBs and combined HAV IgG and anti-HBs						
	No of tested HAV	No (%) of positive HAV IgG	95% CI	P value*	No of tested anti-HBs	No (%) of positive anti-HBs	95% CI	P value	No (%) of positive HAV IgG anti-HBs	95% CI	P value
**Total**	2577	1919 (74.5)	72.7 to 76.1		2551	1967 (77.1)	75.4 to 78.7		1562 (61.2)	59.3 to 63.1	
Clinic group											
TDL North London	450	377 (83.8)	80.1 to 86.9	<0.001	443	341 (77.0)	72.8 to 80.7	<0.001	304 (68.6)	64.2 to 72.8	<0.001
Homerton Hospital	566	401 (70.9)	67.0 to 74.4		564	443 (78.6)	75.0 to 81.7		341 (60.5)	56.4 to 64.4	
St Mary’s Hospital	467	395 (84.6)	81.0 to 87.6		466	390 (83.7)	80.1 to 86.8		335 (71.9)	67.6 to 75.8	
St Thomas’ Hospital	643	477 (74.2)	70.7 to 77.4		628	466 (74.2)	70.6 to 77.5		372 (59.2)	55.3 to 63.0	
Leeds Sexual Health	451	269 (59.7)	55.1 to 64.1		450	327 (72.7)	68.4 to 76.6		210 (46.7)	42.1 to 51.3	
Age group											
18–25	525	320 (61.0)	56.7 to 65.0	<0.001	515	321 (62.3)	58.1 to 66.4	<0.001	219 (42.5)	38.3 to 46.8	<0.001
26–35	979	693 (70.8)	67.9 to 73.5		970	780 (80.4)	77.8 to 82.8		587 (60.5)	57.4 to 63.5	
36–45	600	497 (82.8)	79.6 to 85.6		596	499 (83.7)	80.5 to 86.5		425 (71.3)	67.5 to 74.8	
46 and over	473	409 (86.5)	83.1 to 89.3		470	367 (78.1)	74.1 to 81.6		470 (70.4)	99.2 to 100.0	
Region of birth											
UK	1363	1011 (74.2)	71.8 to 76.4	<0.001	1348	986 (73.2)	70.7 to 75.4	<0.001	791 (58.7)	56.0 to 61.3	0.10
Africa	77	73 (94.8)	87.4 to 98.0		77	64 (83.1)	73.2 to 89.9		61 (79.2)	68.9 to 86.8	
Americas	230	174 (75.7)	69.7 to 80.7		226	181 (80.1)	74.4 to 84.8		146 (64.6)	58.2 to 70.5	
Eastern Mediterranean	71	59 (83.1)	72.7 to 90.1		71	54 (76.1)	65.0 to 84.5		46 (64.8)	53.2 to 74.9	
Europe	565	382 (67.6)	63.6 to 71.3		560	459 (82.0)	78.6 to 84.9		329 (58.8)	54.6 to 62.8	
South-East Asia	47	37 (78.7)	65.1 to 88.0		47	38 (80.9)	67.5 to 89.6		31 (66.0)	51.7 to 77.8	
Western Pacific	132	109 (82.6)	75.2 to 88.1		130	113 (86.9)	80.1 to 91.7		95 (73.1)	64.9 to 80.0	
Unknown	92	74 (80.4)	71.2 to 87.3		92	72 (78.3)	68.8 to 85.5		63 (68.5)	58.4 to 77.1	
History of STI in past year											
No	1889	1359 (71.9)	69.9 to 73.9	<0.001	1867	1392 (74.6)	72.5 to 76.5	<0.001	1081 (57.9)	55.6 to 60.1	<0.001
Yes	688	560 (81.4)	78.3 to 84.1		684	575 (84.1)	81.1 to 86.6		481 (70.3)	66.8 to 73.6	
HIV status											
Negative	2228	1609 (72.2)	70.3 to 74.0	<0.001	2202	1671 (75.9)	74.1 to 77.6	<0.001	1295 (58.8)	56.7 to 60.8	<0.001
Positive	349	310 (88.8)	85.1 to 91.7		349	296 (84.8)	80.7 to 88.2		267 (76.5)	71.8 to 80.6	

*P values from χ^2^ tests.

HAV, hepatitis A virus; STI, sexually transmitted infection; TDL, The Doctor’s Laboratory.

**Table 3:** Univariable and multivariable regression ORs, hepatitis A IgG positivity by patient characteristics (also adjusted for clinic group)

Characteristic	Number tested	Univariable OR HAV IgG	95% CI	P value*	Multivariable OR HAV IgG	95% CI	P value
**Total**	2577						
Age group							
18–25	525	Ref		<0.001	Ref		<0.001
26–35	979	1.55	1.24 to 1.94		1.5	1.18 to 1.86	
36–45	600	3.09	2.35 to 4.07		2.91	2.17 to 3.90	
46 and over	473	4.09	2.98 to 5.62		3.4	2.44 to 4.75	
HIV status							
Negative	2228	Ref		<0.001			0.170
Positive	349	3.06	2.37 to 2.85		1.82	1.25 to 2.64	
History of STI in past year							
No	1889	Ref		<0.001	Ref		<0.001
Yes	688	1.71	1.37 to 2.12		1.58	1.25 to 2.00	
Country of birth							
UK	1363	Ref		0.418	Not included		
Non-UK	1123	1	0.84 to 1.21				
Unknown	91	1.41	0.83 to 2.40				
Region of birth							
UK	1363	Ref		<0.001	Ref		<0.001
Africa	77	6.35	2.31 to 17.51		4.73	1.68 to 13.31	
Americas	230	1.08	0.78 to 1.50		0.64	0.45 to 0.91	
Eastern Mediterranean	71	1.71	0.91 to 3.22		1.41	0.72 to 2.73	
Europe	565	0.73	0.59 to 0.90		0.56	0.44 to 0.71	
South-East Asia	47	1.29	0.63 to 2.62		0.9	0.43 to 1.91	
Western Pacific	132	1.65	1.04 to 2.63		1.44	0.89 to 2.35	
Unknown	92	1.43	0.84 to 2.43		1.26	0.72 to 2.20	

*Global p values representing the overall impact of the variable on the model.

HAV, hepatitis A virus; STI, sexually transmitted infection.

### Weighted anti-HBs seroprevalence and associated risk factors

The weighted seroprevalence of anti-HBs was 76.4%. Overall, 77.1% (1967 of 2551) of samples were anti-HBs positive, indicating immunity due to vaccination, or to a lesser extent, past exposure to HBV infection (table 2). Seroprevalence of HBV immunity varied by clinic group, ranging from 72.7% in Leeds to 83.7% in London St Mary’s Hospital (global p<0.001). In multivariable analysis, by age group, adjusted seroprevalence of HBV immunity was highest in those aged 36–45 (OR 2.91, 2.17 to 3.90 compared with the 18–25 years age group) (table 4). By region of birth, seroprevalence of HBV immunity was highest in those born in the Western Pacific region (OR 2.40, 1.41 to 4.11) followed by those born in the rest of Europe (OR 1.40, 1.08 to 1.82 compared with the UK). Seroprevalence of HBV immunity was higher in those with an STI in the past year than those without (OR 1.61, 1.27 to 2.05).

**Table 4:** Univariable and multivariable regression ORs, hepatitis B surface antibody (anti-HBs) positivity by patient characteristics (also adjusted for clinic)

Characteristic	Number tested	Univariable OR anti-HBs	95% CI	P^2^	Multivariable OR anti-HBs	95% CI	P^2^
**Total**	2551						
Age group							
18–25	515	Ref		<0.001			<0.001
26–35	970	2.48	1.95 to 3.15		2.32	1.81 to 2.97	
36–45	596	3.11	2.35 to 4.12		2.91	2.17 to 3.90	
46 and over	470	2.15	1.62 to 2.85		2.05	1.52 to 2.76	
HIV status							
Negative	2202	Ref		<0.001			0.170
Positive	349	1.77	1.30 to 2.42		1.26	0.90 to 1.77	
History of STI in past year							
No	1867	Ref		<0.001			<0.001
Yes	684	1.80	1.43 to 2.26		1.61	1.27 to 2.05	
Country of birth					Not included		
UK	1348	Ref		<0.001			
Non-UK	1112	1.65	1.36 to 2.01				
Unknown	91	1.30	0.78 to 2.17				
Region of birth							
UK	1348	Ref		<0.001			0.020
Africa	77	1.81	0.98 to 3.32		1.43	0.77 to 2.65	
Americas	226	1.48	1.04 to 2.09		1.14	0.79 to 1.64	
Eastern Mediterranean	71	1.17	0.67 to 2.04		0.97	0.54 to 1.72	
Europe	560	1.67	1.31 to 2.14		1.40	1.08 to 1.81	
South-East Asia	47	1.55	0.74 to 3.24		1.28	0.61 to 2.73	
Western Pacific	130	2.44	1.45 to 4.12		2.40	1.41 to 4.11	
Unknown	92	1.32	0.79 to 2.20		1.05	0.61 to 1.80	

STI, sexually transmitted infection.

### Combined HAV and HBV seroprevalence

Overall, 43.8% (1119 of 2544, 42.0–45.8) of the samples had combined immunity for HAV and HBV (table 3). Distribution of combined immunity was overall similar to the distributions of the individual markers but did not vary significantly by region of birth (p=0.1).

## Discussion

### Main findings

Collectively, our findings indicate high prevalence of immunity to HAV (74.5%) and HBV (77.1%) in GBMSM attending SHS located mainly in London, England. Combined immunity to both infections was 43.8%. Weighted seroprevalence was similar to unweighted at 74.3% for HAV and 76.4% for HBV. Seroprevalence of HAV immunity was above the 70% threshold estimated by an Australian study to be the critical level of immunity to prevent sustained outbreaks in the GBMSM community.^20^

For both infections, the proportion that was immune varied by clinic group and was higher among those known to be living with HIV or with a history of STI in the past year, and higher in older age groups compared with the 18–25 years age group. Seroprevalence of HAV immunity was higher among those born in the African region and lower among those born in the rest of Europe and the Americas compared with the UK, whereas seroprevalence of HBV immunity was higher in those born in the Western Pacific region and in the rest of Europe compared with the UK. Our sample appears to be representative of the GBMSM attending the participating clinics; although there was some bias towards lower-risk individuals in our sample as defined by HIV status and past STI, weighted seroprevalence estimates to account for variations in demographics and risk indicators between our sample and other GBMSM attendees were very similar to unweighted.

### Limitations

Our study recruited nine SHS, eight of which were in London. Included clinics represent a small sample of SHS in England and may not be representative, particularly as access to SHS and service provision in London may be different to UK settings outside London. Our samples were collected after the start of the outbreak, so we do not have true baseline data on HAV immunity before the outbreak and the updated BASHH vaccine recommendations in 2017.^5^ A further limitation is that our study does not provide seroprevalence for GBMSM who do not attend SHS and may not capture information on *HI*-MSM if they do not identify as MSM when they attend SHS, both of which are needed to understand the risk of future outbreaks. Another limitation is that our study does not include information on HIV pre-exposure prophylaxis (HIV-PrEP) use among our sample. This would be a helpful addition for future studies as those using HIV-PrEP are engaged with SHS and so likely to access vaccinations more readily than those attending SHS for testing alone.

Interpretation of serology results is limited by the sensitivity and specificity of the assays used, and there is potential for misclassification of immunity depending on the cut-offs used. It is possible that some HBV-immunised GBMSM were misclassified as susceptible.

Despite these limitations, the strength of this study lies in serological assessment of immunity over studies that rely on self-reported measures alone, which have been shown to be limited.^21^

### Public health implications

As HAV and HBV are no longer endemic in the UK and many other high-income countries, it is likely that most immunity in our sample was because of vaccination, but some will be caused by exposure in countries of origin and during the recent outbreaks of HAV. Similar to other studies, both HAV and HBV immunity were lower in younger adults (18–25 years old) at 61% and 62%, respectively. Higher immunity among older GBMSM is likely due to a combination of increased exposure to infection and more opportunities for vaccination over time. Immunity varied by country of origin, likely due to variations in endemicity and universal vaccination programmes; for HBV, immunity was higher in those born in the Western Pacific region, where the prevalence of HBV infection is high, and the rest of Europe, where several countries have had universal HBV vaccination programmes running for longer than in the UK, where universal infant vaccination had started in 2017 when this study began.

HBV immunity was much higher than the 30% found among users of a home self-sampling HIV testing service, highlighting the need to consider how to provide access to vaccinations to higher-risk individuals who do not access on-site SHS.^22^

Immunity varied by clinic group and for HAV, was lower in Leeds than in London clinics. This is likely due to a combination of time and geographical factors; the Leeds samples were the only samples taken before the updated BASHH vaccine recommendations in 2017, and because the outbreak peaked in London, there was potentially a greater impetus for London clinics to increase vaccination compared with clinics elsewhere.^6^

Immunity was associated with HIV positive status and history of STI, both of which are associated with sexual behaviours also linked to increased risk of viral hepatitis infection, suggesting targeting of vaccination to GBMSM at higher risk of infection with these viral hepatitides.^7^ GBMSM living with HIV may also have been offered vaccination due to national guidelines informing routine HIV clinical care.^23^ This suggests that recommendations for universal GBMSM HAV and HBV vaccination in SHS are not always followed.

While this serosurvey provides a snapshot of HAV and HBV immunity in 2017–2018, periodical monitoring of serological immunity in GBMSM is needed to ensure the selective immunisation programme is achieving its objectives and gives early warning of falls in immunity that may allow outbreaks to occur. Strategies to improve vaccine offer and uptake in all GBMSM are needed in England to achieve and maintain herd immunity. In addition, concerted efforts and formative research are needed to explore the barriers to vaccine offer and uptake for some GBMSM accessing SHS, improve vaccine signposting and explore alternative ways of offering vaccinations to those who choose online self-sampling rather than traditional services. The recent HAV and HBV outbreaks in GBMSM and *HI*-MSM, respectively, serve as a reminder that there are subgroups of GBMSM who do not routinely access SHS and/or do not consider themselves at risk, miss out on vaccination and contribute to a growing pool of susceptible persons in which outbreaks can become established.

## Conclusions

Our study provides a baseline seroprevalence from which to monitor serial immunity to HBV and HAV in GBMSM accessing SHS, as an adjunct to recorded vaccine coverage while these methods are being developed. Levels of immunity in this study were reasonably high overall at 75% and 77% for HAV and HBV, noting samples were taken after recent widespread outbreaks and related vaccination campaigns. Older age, history of STI in the past year and region of birth were associated with immunity. High vaccine coverage in all GBMSM should be maintained to prevent further outbreaks.

## Data Availability

Data are available upon reasonable request. The datasets used and analysed during this study are available from the corresponding author on reasonable request.
