# Supplementary material for: Seroprevalence of immunity to hepatitis A and hepatitis B among gay, bisexual and other men who have sex with men (GBMSM) attending sexual health clinics in London and Leeds, England, 2017–2018
Source: Sex Transm Infect. 2024 Jun 26;100(5):281–7. doi: 10.1136/sextrans-2024-056134 (PMC11287524; doi:10.1136/sextrans-2024-056134)
Supplement: Supplementary data [file sextrans-2024-056134supp001.pdf]

Supplementary information

Supplementary Table 1: Definitions of GUMCAD variables used in the analysis

Variable	Definition
History of STI in past year	Diagnosed with a new STI (excluding HIV) in the year prior to attendance (1-365 days)
HIV diagnosed	An individual is defined in GUMCAD as living with diagnosed HIV for all attendances following either a new HIV diagnosis code or an attendance for HIV related care
Country of birth / WHO region of birth	Patients' country of birth as self-reported by the patient at registration. WHO region of birth derived from this using the 6 WHO regions (1).
HAV diagnosis	Recorded diagnosis of acute HAV (defined as detection of hepatitis A virus specific IgM antibodies)
HAV vaccine (1st)	First dose of a new hepatitis A vaccination course
HAV vaccine (2nd)	Second dose of a hepatitis A vaccination course (including those who may have received an earlier dose at another sexual health service);
HAV immune	HAV immunity includes those who have i) natural immunity – via self-reported or documented history of hepatitis A infection, ii) vaccine-induced immunity – via documented history of 2 doses of monovalent or 3 doses of bivalent vaccine
HBV vaccine (1st)	First dose of a new hepatitis B vaccination course (including those who may have been previously vaccinated at any sexual health service but are now starting a new vaccination course).
HBV vaccine (2nd, 3rd, 4th)	Second, third or fourth dose of a HBV vaccination course (including those who may have received an earlier dose at another sexual health service);
HBV vaccine booster	HBV vaccination booster dose (including those who may have been vaccinated at another sexual health service)
HBV immune	HBV immunity includes those who have: i) natural immunity – via self-reported or documented history of hepatitis B infection, ii) vaccinated immunity – via documented history of 3 doses of vaccine
HBV diagnosed	Recorded first diagnosis of HBV (first diagnosis of viral hepatitis B – HBsAG antigen positive)

Supplementary Table 2: Sample size calculations

BBV	Age group	Estimated prevalence	Sample size	Estimated numerator	LCI	UCI
HAV[1]	<40	20.0%	260	52	15.6%	25.3%
	40-59	46.4%	250	116	40.3%	52.6%
	60+	73.5%	150	110	65.9%	79.9%
HBV[2]	18-24	78.8%	60	47	66.9%	87.3%
	25-29	50.0%	150	75	42.1%	57.9%
	30-39	31.3%	150	47	24.4%	39.1%
	40+	24.0%	100	24	16.7%	33.2%

1. HAV seroprevalence estimates taken from Morris et al 2002 (2) and age groups as initially planned for this study before sample collection.
2. HBV seroprevalence estimates taken from Pitasi et al 2014 (3), age groups as shown in that study.

Supplementary Table 3. Sample characteristics vs other GBMSM clinic attendees

		Sample			Other GBMSM clinic attendees			
**Characteristic**		**Number**	**% of total**	**95% CI**	**Number**	**% of total**	**95% CI**	**P (difference between sample and comparison group (Chi^2))**
**TOTAL**		2577	**100**		5939	**100**		
Clinic group	TDL North London	450	**17.5**		539	**9.1**		
	Homerton Hospital	566	**22.0**		1441	**24.3**		
	St Mary's Hospital	467	**18.1**		733	**12.3**		
	St Thomas' Hospital	643	**25.0**		2711	**45.6**		
	Leeds Sexual Health	451	**17.5**		515	**8.7**		
Age group	***All***							
	18-25	525	**20.4**	18.9-22.0	1100	**18.5**	17.6-19.5	0.014
	26-35	979	**38.0**	36.1-39.9	2406	**40.5**	39.3-41.8	
	36-45	600	**23.3**	21.7-25.0	1451	**24.4**	23.4-25.5	
	46 and over	473	**18.4**	16.9-19.9	982	**16.5**	15.6-17.5	
	TDL North London							
	18-25	114	**25.3**	21.5-29.5	118	**21.9**	18.6-25.6	0.014
	26-35	174	**38.7**	34.3-43.2	172	**31.9**	28.1-36.0	
	36-45	85	**18.9**	15.5-22.8	130	**24.1**	20.7-27.9	
	46 and over	77	**17.1**	13.9-20.9	119	**22.1**	18.8-25.8	
	Homerton Hospital							
	18-25	107	**18.9**	15.9-22.3	267	**18.5**	16.6-20.6	0.027
	26-35	290	**51.2**	47.1-55.3	666	**46.2**	43.7-48.8	
	36-45	121	**21.4**	18.2-24.9	322	**22.3**	20.3-24.6	

	46 and over	48	**8.5**	6.5-11.1	186	**12.9**	11.3-14.7	
	St Mary's Hospital							
	18-25	59	**12.6**	9.9-16.0	95	**13.0**	10.7-15.6	0.326
	26-35	167	**35.8**	31.5-40.2	225	**30.7**	27.5-34.1	
	36-45	121	**25.9**	22.1-30.1	210	**28.6**	25.5-32.0	
	46 and over	120	**25.7**	21.9-29.8	203	**27.7**	24.6-31.0	
	St Thomas' Hospital							
	18-25	100	**15.6**	13.0-18.6	484	**17.9**	16.5-19.3	<0.0001
	26-35	181	**28.1**	24.8-31.7	1155	**42.6**	40.8-44.5	
	36-45	197	**30.6**	27.2-34.3	695	**25.6**	24.0-27.3	
	46 and over	165	**25.7**	22.4-29.2	377	**13.9**	12.7-15.3	
	Leeds Sexual Health							
	18-25	145	**32.2**	28.0-36.6	136	**26.4**	22.8-30.4	0.092
	26-35	167	**37.0**	32.7-41.6	188	**36.5**	32.5-40.7	
	36-45	76	**16.9**	13.7-20.6	94	**18.3**	15.2-21.8	
	46 and over	63	**14.0**	11.1-17.5	97	**18.8**	15.7-22.4	
History of STI in past year	***All***							
	No	1889	**73.3**	71.6-75.0	4045	**68.1**	66.9-69.3	<0.0001
	Yes	688	**26.7**	25.0-28.4	1894	**31.9**	30.7-33.1	
	TDL North London							
	No	351	**78.0**	73.9-81.6	385	**71.4**	67.5-75.1	0.018
	Yes	99	**22.0**	18.4-26.1	154	**28.6**	24.9-32.5	
	Homerton Hospital							
	No	389	**68.7**	64.8-72.4	1007	**69.9**	67.5-72.2	0.613
	Yes	177	**31.3**	27.6-35.2	434	**30.1**	27.8-32.5	
	St Mary's Hospital							
	No	268	**57.4**	52.9-61.8	422	**57.6**	54.0-61.1	0.95
	Yes	199	**42.6**	38.2-47.1	311	**42.4**	38.9-46.0	

	St Thomas' Hospital							
	No	530	**82.4**	79.3-85.2	1853	**68.4**	66.6-70.1	<0.0001
	Yes	113	**17.6**	14.8-20.7	858	**31.6**	29.9-33.4	
	Leeds Sexual Health							
	No	351	**77.8**	73.8-81.4	378	**73.4**	69.4-77.0	0.11
	Yes	100	**22.2**	18.6-26.2	137	**26.6**	23.0-30.6	
HIV status	***All***							
	Negative	2228	**86.5**	85.1-87.7	4494	**75.7**	74.6-76.7	<0.0001
	Positive	349	**13.5**	12.3-14.9	1445	**24.3**	23.3-25.4	
	TDL North London							
	Negative	361	**80.2**	76.3-83.6	337	**62.5**	58.4-66.5	<0.0001
	Positive	89	**19.8**	16.4-23.7	202	**37.5**	33.5-41.6	
	Homerton Hospital							
	Negative	546	**96.5**	94.6-97.7	1164	**80.8**	78.7-82.7	<0.0001
	Positive	20	**3.5**	2.3-5.4	277	**19.2**	17.3-21.3	
	St Mary's Hospital							
	Negative	323	**69.2**	64.8-73.2	526	**71.8**	68.4-74.9	0.335
	Positive	144	**30.8**	26.8-35.2	207	**28.2**	25.1-31.6	
	St Thomas' Hospital							
	Negative	590	**91.8**	89.4-93.6	2022	**74.6**	72.9-76.2	<0.0001
	Positive	53	**8.2**	6.4-10.6	689	**25.4**	23.8-27.1	
	Leeds Sexual Health							
	Negative	408	**90.5**	87.4-92.8	445	**86.4**	83.2-89.1	0.05
	Positive	43	**9.5**	7.2-12.6	70	**13.6**	10.9-16.8	
Country of birth	***All***							
	UK	1363	**52.9**	51.0-54.8	3233	**54.4**	53.2-55.7	0.229
	non-UK	1123	**43.6**	41.7-45.5	2475	**41.7**	40.4-42.9	

	unknown	91	**3.5**	2.9-4.3	231	**3.9**	3.4-4.4	
	TDL North London							
	UK	241	**53.6**	48.9-58.1	282	**52.3**	48.1-56.5	0.618
	non-UK	209	**46.4**	41.9-51.1	256	**47.5**	43.3-51.7	
	unknown	0	**0.0**	0.0-0.8	1	**0.2**	0.0-1.0	
	Homerton Hospital							
	UK	251	**44.3**	40.3-48.5	617	**42.8**	40.3-45.4	0.219
	non-UK	254	**44.9**	40.8-49.0	627	**43.5**	41.0-46.1	
	unknown	61	**10.8**	8.5-13.6	197	**13.7**	12.0-15.5	
	St Mary's Hospital							
	UK	160	**34.3**	30.1-38.7	262	**35.7**	32.4-39.3	0.32
	non-UK	291	**62.3**	57.8-66.6	456	**62.2**	58.6-65.6	
	unknown	16	**3.4**	2.1-5.5	15	**2.0**	1.2-3.3	
	St Thomas' Hospital							
	UK	364	**56.6**	52.8-60.4	1665	**61.4**	59.6-63.2	0.081
	non-UK	277	**43.1**	39.3-46.9	1039	**38.3**	36.5-40.2	
	unknown	2	**0.3**	0.1-1.1	7	**0.3**	0.1-0.5	
	Leeds Sexual Health							
	UK	347	**76.9**	72.8-80.6	407	**79.0**	75.3-82.3	0.7
	non-UK	92	**20.4**	16.9-24.4	97	**18.8**	15.7-22.4	
	unknown	12	**2.7**	1.5-4.6	11	**2.1**	1.2-3.8	
Region of birth	***All***							
	UK	1363	**52.9**	51.0-54.8	3233	**54.4**	53.2-55.7	0.574
	Africa	77	**3.0**	2.4-3.7	187	**3.1**	2.7-3.6	
	Americas	230	**8.9**	7.9-10.1	505	**8.5**	7.8-9.2	
	Eastern Mediterranean	71	**2.8**	2.2-3.5	151	**2.5**	2.2-3.0	
	Europe	565	**21.9**	20.4-23.6	1270	**21.4**	20.4-22.4	

	South-East Asia	47	**1.8**	1.4-2.4	80	**1.3**	1.1-1.7	
	Western Pacific	132	**5.1**	4.3-6.0	281	**4.7**	4.2-5.3	
	Unknown	92	**3.6**	2.9-4.4	232	**3.9**	3.4-4.4	
HAV Vaccine (1st)								
	No	2224	**86.3**	84.9-87.6	5266	**88.7**	87.8-89.4	0.002
	Yes	353	**13.7**	12.4-15.1	673	**11.3**	10.6-12.2	
HAV Vaccine (2nd)								
	No	2495	**96.8**	96.1-97.4	5693	**95.9**	95.3-96.3	0.034
	Yes	82	**3.2**	2.6-3.9	246	**4.1**	3.7-4.7	
HAV vaccine (any)								
	No	2192	**85.1**	83.6-86.4	5139	**86.5**	85.6-87.4	0.072
	Yes	385	**14.9**	13.6-16.4	800	**13.5**	12.6-14.4	
HAV immune								
	No	2022	**78.5**	76.8-80.0	5174	**87.1**	86.2-87.9	<0.0001
	Yes	555	**21.5**	20.0-23.2	765	**12.9**	12.1-13.8	
HBV Vaccine (1st)								
	No	2129	**82.6**	81.1-84.0	5087	**85.7**	84.7-86.5	<0.0001
	Yes	448	**17.4**	16.0-18.9	852	**14.3**	13.5-15.3	
HBV Vaccine (2nd)								
	No	2316	**89.9**	88.6-91.0	5360	**90.3**	89.5-91.0	0.59
	Yes	261	**10.1**	9.0-11.4	579	**9.7**	9.0-10.5	
HBV Vaccine (3rd)								
	No	2345	**91.0**	89.8-92.0	5488	**92.4**	91.7-93.1	0.028
	Yes	232	**9.0**	8.0-10.2	451	**7.6**	6.9-8.3	
HBV Vaccine (4th)								
	No	2550	**99.0**	98.5-99.3	5894	**99.2**	99.0-99.4	0.179
	Yes	27	**1.0**	0.7-1.5	45	**0.8**	0.6-1.0	

HBV Vaccine (any)								
	No	1855	**72.0**	70.2-73.7	4496	**75.7**	74.6-76.8	<0.0001
	Yes	722	**28.0**	26.3-29.8	1443	**24.3**	23.2-25.4	
HBV Booster								
	No	2382	**92.4**	91.3-93.4	5518	**92.9**	92.2-93.5	0.434
	Yes	195	**7.6**	6.6-8.7	421	**7.1**	6.5-7.8	
HBV immune								
	No	1955	**75.9**	74.2-77.5	5102	**85.9**	85.0-86.8	<0.0001
	Yes	622	**24.1**	22.5-25.8	837	**14.1**	13.2-15.0	

Supplementary table 4: Positivity by age group and clinic

**Characteristic**	**HAV IgG**				**Anti-HBs and combined HAV IgG and anti-HBs**						
	**No. tested HAV**	**No. (%) positive HAV IgG**	**95% CI**	**P**	**No. tested anti-HBs**	**No. (%) positive anti-HBs**	**95% CI**	**P**	**No. (%) positive HAV IgG and anti-HBs**	**95% CI**	**P**
**TOTAL**	2577	1919 (74.5)	72.7-76.1		2551	1967 (77.0)	75.4-78.7		1562 (61.2)	59.3-63.1	
Clinic											
TDL North London	450	377 (83.8)	80.1-86.9	<0.0001	443	341 (77.0)	72.8-80.7	0.001	304 (68.6)	64.2-72.8	<0.0001
Homerton Hospital	566	401 (70.9)	67.0-74.4		564	443 (78.6)	75.0-81.7		341 (60.5)	56.4-64.4	
St Mary's Hospital	467	395 (84.6)	81.0-87.6		466	390 (83.7)	80.1-86.8		335 (71.9)	67.6-75.8	
St Thomas' Hospital	643	477 (74.2)	70.7-77.4		628	466 (74.2)	70.6-77.5		372 (59.2)	55.3-63.0	
Leeds Sexual Health	451	269 (59.7)	55.1-64.1		450	327 (72.7)	68.4-76.6		210 (46.7)	42.1-51.3	
Age group											
18-25	525	320 (61.0)	56.7-65.0	<0.0001	515	321 (62.3)	58.1-66.4	<0.0001	219 (42.5)	38.3-46.8	<0.0001
26-35	979	693 (70.8)	67.9-73.5		970	780 (80.4)	77.8-82.8		587 (60.5)	57.4-63.5	
36-45	600	497 (82.8)	79.6-85.6		596	499 (83.7)	80.5-86.5		425 (71.3)	67.5-74.8	
46 and over	473	409 (86.5)	83.1-89.3		470	367 (78.1)	74.1-81.6		470 (70.4)	99.2-100.0	
Age groups by clinic											
TDL North London											
18-25	114	90 (79.0)	70.6-85.4	0.179	112	72 (64.3)	55.1-72.6	0.001	61 (54.5)	45.2-63.4	0.001
26-35	174	145 (83.3)	77.1-88.1		169	133 (78.7)	71.9-84.2		118 (69.8)	62.5-76.2	
36-45	85	77 (90.6)	82.5-95.2		85	75 (88.2)	79.7-93.5		69 (81.2)	71.6-88.1	
46 and over	77	65 (84.4)	74.7-90.9		77	61 (79.2)	68.9-86.8		56 (72.7)	61.9-81.4	
Homerton Hospital											
18-25	107	62 (57.9)	48.5-66.9	0.001	106	61 (57.6)	48.0-66.5	<0.0001	42 (39.6)	30.8-49.1	<0.0001
26-35	290	204 (70.3)	64.8-75.3		289	244 (84.4)	79.8-88.2		184 (63.7)	58.0-69.0	
36-45	121	93 (76.9)	68.6-83.5		121	98 (81.0)	73.1-87.0		79 (65.3)	56.5-73.2	
46 and over	48	42 (87.5)	75.3-94.1		48	40 (83.3)	70.4-91.3		36 (75.0)	61.2-85.1	

St Mary's Hospital											
18-25	59	42 (71.2)	58.6-81.2	<0.0001	58	44 (75.9)	63.5-85.0	0.028	32 (55.2)	42.5-67.3	<0.0001
26-35	167	134 (80.2)	73.6-85.6		167	136 (81.4)	74.9-86.6		111 (66.5)	59.0-73.2	
36-45	121	109 (90.1)	83.5-94.2		121	111 (91.7)	85.5-95.4		99 (81.8)	74.0-87.7	
46 and over	120	110 (91.7)	85.3-95.4		120	99 (82.5)	74.7-88.3		93 (77.5)	69.2-84.1	
St Thomas' Hospital											
18-25	100	57 (57.00	47.2-66.3	<0.0001	95	48 (50.5)	40.6-60.4	<0.0001	33 (34.7)	25.9-44.7	<0.0001
26-35	181	112 (61.9)	54.6-68.6		178	135 (75.8)	69.1-81.5		89 (50.0)	42.7-57.3	
36-45	197	166 (84.3)	78.5-88.7		193	158 (81.9)	75.8-86.7		139 (72.0)	65.3-77.9	
46 and over	165	142 (86.1)	80.0-90.5		162	125 (77.2)	70.1-83.0		111 (68.5)	61.0-75.2	
Leeds Sexual Health											
18-25	145	69 (47.6)	39.6-55.7	<0.0001	144	96 (66.7)	58.6-73.8	0.061	51 (35.4)	28.1-43.5	0.011
26-35	167	98 (58.7)	51.1-65.9		167	132 (79.0)	72.3-84.5		85 (50.9)	43.4-58.4	
36-45	76	52 (68.4)	57.3-77.8		76	57 (75.00	64.2-83.4		39 (51.3)	40.3-62.2	
46 and over	63	50 (79.4)	67.8-87.5		63	42 (66.7)	54.4-77.1		35 (55.6)	43.3-67.2	

1. World Health Organisation. Countries [Available from: https://www.who.int/countries.
2. Morris MC, Gay NJ, Hesketh LM*, et al.* The changing epidemiological pattern of hepatitis A in England and Wales. Epidemiology and infection. 2002;128(3):457-63.

3. Pitasi MA, Bingham TA, Sey EK*, et al.* Hepatitis B Virus (HBV) Infection, Immunity and Susceptibility Among Men Who Have Sex with Men (MSM), Los Angeles County, USA. AIDS and Behavior. 2014;18(3):248-55. Available from: https://doi.org/10.1007/s10461-013-0670-2.
